# New Perspectives in the Renin-Angiotensin-Aldosterone System (RAAS) II: Albumin Suppresses Angiotensin Converting Enzyme (ACE) Activity in Human

**DOI:** 10.1371/journal.pone.0087844

**Published:** 2014-04-01

**Authors:** Miklós Fagyas, Katalin Úri, Ivetta M. Siket, Gábor Á. Fülöp, Viktória Csató, Andrea Daragó, Judit Boczán, Emese Bányai, István Elek Szentkirályi, Tamás Miklós Maros, Tamás Szerafin, István Édes, Zoltán Papp, Attila Tóth

**Affiliations:** 1 Division of Clinical Physiology, Institute of Cardiology, University of Debrecen, Debrecen, Hungary; 2 Department of Neurology, University of Debrecen, Debrecen, Hungary; 3 Institute of Internal Medicine, Division of Nephrology, University of Debrecen, Debrecen, Hungary; 4 Department of Cardiac Surgery, Institute of Cardiology, University of Debrecen, Debrecen, Hungary; School of Pharmacy, Texas Tech University HSC, United States of America

## Abstract

About 8% of the adult population is taking angiotensin-converting enzyme (ACE) inhibitors to treat cardiovascular disease including hypertension, myocardial infarction and heart failure. These drugs decrease mortality by up to one-fifth in these patients. We and others have reported previously that endogenous inhibitory substances suppress serum ACE activity, *in vivo*, similarly to the ACE inhibitor drugs. Here we have made an effort to identify this endogenous ACE inhibitor substance. ACE was crosslinked with interacting proteins in human sera. The crosslinked products were immunoprecipitated and subjected to Western blot. One of the crosslinked products was recognized by both anti-ACE and anti-HSA (human serum albumin) antibodies. Direct ACE-HSA interaction was confirmed by binding assays using purified ACE and HSA. HSA inhibited human purified (circulating) and human recombinant ACE with potencies (IC_50_) of 5.7±0.7 and 9.5±1.1 mg/mL, respectively. Effects of HSA on the tissue bound native ACE were tested on human saphenous vein samples. Angiotensin I evoked vasoconstriction was inhibited by HSA in this vascular tissue (maximal force with HSA: 6.14±1.34 mN, without HSA: 13.54±2.63 mN), while HSA was without effects on angiotensin II mediated constrictions (maximal force with HSA: 18.73±2.17 mN, without HSA: 19.22±3.50 mN). The main finding of this study is that HSA was identified as a potent physiological inhibitor of the ACE. The enzymatic activity of ACE appears to be almost completely suppressed by HSA when it is present in its physiological concentration. These data suggest that angiotensin I conversion is limited by low physiological ACE activities, *in vivo*.

## Introduction

The renin-angiotensin-aldosterone system (RAAS) is an important regulator of blood pressure and salt-water homeostasis [1]. One of the elements of this system is the angiontensin converting enzyme. It is a zinc-metalloendodipeptidase which catalyzes the cleavage of angiotensin I to angiotensin II, and the metabolism of other peptides such as bradykinin [2]. ACE has two isoenzymes: a somatic and a testicular form [3]. The somatic form of ACE is found on the membrane surface of various cells and it can be released into the circulation by ACE secretase [4], [5].

Inhibition of ACE is beneficial in cardiovascular diseases [6]–[8]. The latest therapeutic guidelines have incorporated all these therapeutic benefits of ACE inhibition [9]–[14]. ACE inhibitors are an important components of a hypothetical polypill proposed to reduce cardiovascular disease by 80% [15]. Importance of ACE inhibition may also be highlighted by the fact that there are almost 47,000 hits in the Medline for “ACE inhibitor”. The vast majority of these articles are presenting various features of exogenous ACE inhibition in animal models and clinical trials.

Interestingly, ACE activity may also be inhibited by endogenous inhibiting factors, although these endogenous antagonists were generally neglected in the past decades, in spite of the clinical success of pharmacological ACE inhibitors. The first reports regarding these endogenous antagonists dates back to 1979, when Ryan et al. reported that small (<10 kDa) molecular weight molecules can inhibit ACE [16]. Several more inhibitors has been suggested afterward [17]–[20]. Some efforts were also made to isolate inhibitory molecules associating with ACE in the rat lung [21] or in human sera [22]. In particular, Lieberman reported an endogenous ACE inhibitor with an apparent molecular mass of >50 kDa in 1986 (and gained 16 citations so far) [20], Ikemoto and co-workers reported an endogenous inhibitor (>10 kDa) in 1989 (22 citations) [19].

In a recent work we have confirmed the existence of an endogenous ACE inhibitor in human sera [23]. Our measurements suggested that it is a serum protein with an apparent molecular weight of 50–100 kDa [23]. Here we have made an effort to identify this ACE inhibitory endogenous substance.

## Methods

### Ethical approval

All of the studies were approved by the Regional and Institutional Ethics Committee, Medical and Health Science Center, University of Debrecen, (UDMHSC REC/IEC number: 2894–2008) and by the Medical Research Council of Hungary. All of the individuals involved gave their written informed consent.

### Blood sample collection, serum isolation

Blood samples were collected from volunteers by using a standard aseptic technique. Native blood was incubated for 60 minutes at room temperature, serum fractions (separated by centrifugation at 1,500 *g* for 15 min) were stored at −20°C until further experiments.

### ACE activity measurement using spectrophotometric assay

The basis of the ACE activity measurement was originally described by Beneteau et al. [24] and modified by us [23]. The artificial substrate (FAPGG, (N-[3-(2-furyl)acryloyl]-L-phenylalanylglycylglycine; Sigma-Aldrich) was selectively cleaved by serum ACE in a reaction mixture containing 25 mM HEPES buffer (N-2-hydroxyethylpiperazine-N-2-ethanesulfonic acid), 0.5 mM FAPGG, 300 mM NaCl, at pH 8.2. The activity measurements were performed in 96-well plates (Greiner-Bio One) at 37°C, and the changes in optical density (340 nm) were measured at 5-min interval for at least 90 min with a plate reader (NovoStar plate reader, BMG Labtech). Blank corrected optical density values were plotted as a function of reaction time and fitted by linear regression (GraphPad Prism 5.0). The measurement and the goodness of fitting were accepted, when r^2^ was >0.90. ACE activity was calculated by the equation:

activity = −(S/k)*D,

where S is the rate of observed decrease in optical density (1/min), k is the change in optical density upon the complete cleavage of 1 μmol of FAPGG, and D is the dilution of the serum. ACE activity is given in units where 1 U is equivalent to the cleavage of 1 μmol of FAPGG in 1 min.

### Properties of human serum albumin (HSA)

**Figure 1:**
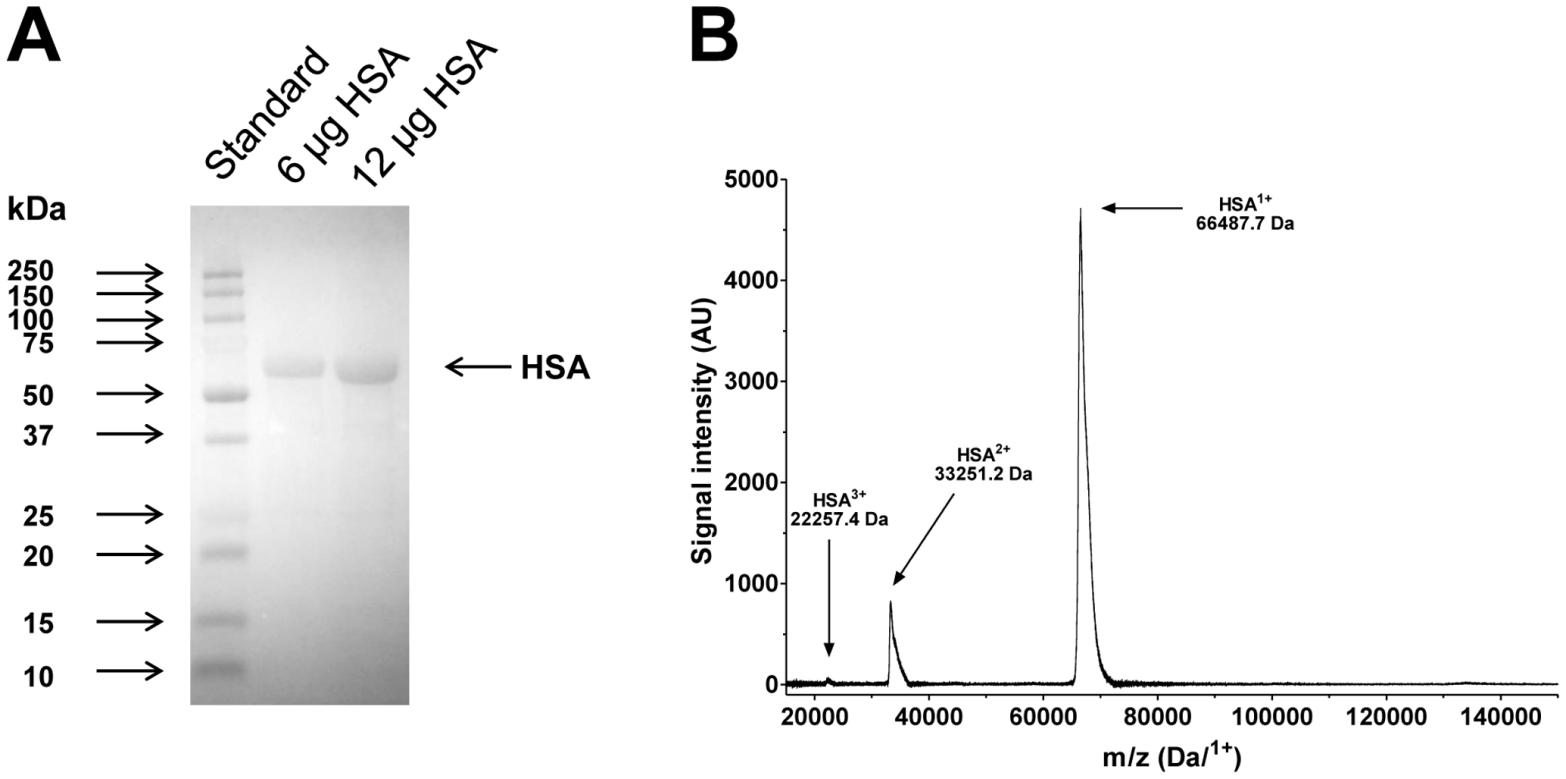
Characteristics of Human serum albumin (HSA). Human serum albumin was obtained from Human BioPlazma, Gödöllő, Hungary. The purity of the HSA was tested by SDS-PAGE first (**A**). There was 6 and 12 μg HSA loaded into the wells. Gel was then stained by Coomassie to visualize proteins. Apparent molecular masses of a set of standard proteins (arrows on the left) and the expected position of the HSA (arrow on the right) are indicated. Mass spectrometric (MALDI-TOF) analysis was also done (**B**). A representative spectrogram is shown on the figure, where the peaks representing the differently ionized HSA molecules are shown.

In some experiments, the ACE activity was measured in the presence of human serum albumin (HSA, Human BioPlazma Manufacturing and Trading). The purity of the HSA preparation was tested by SDS-PAGE (Fig. 1A) and mass spectrometry (Fig. 1B). Both assays showed a highly purified HSA. HSA was also tested for absorbed small molecular weight ACE inhibitors. In these experiments 20 mg/mL HSA was prepared in the buffer used to measure ACE activity with FAPGG substrate. HSA was diluted to 10-fold in each step, and filtered with a membrane with a pore size of 5 kDa. The samples were filtered until the HSA concentration reached the initial 20 mg/mL. The number of filtration cycles were 5, 10 and 15. At the end of the filtration cycles the efficacy of 10 mg/mL HSA was tested on recombinant ACE inhibition using FAPGG substrate. In addition, captopril (1 μM) was also used in a parallel measurement to estimate maximal ACE inhibition.

### Measurement of domain specific ACE activity

Domain specific ACE activity was measured as originally described by Carmona et al. [25] and modified by us [23]. In brief, quenched fluorescent peptide substrates were used, Abz-SDK(Dnp)P-OH (Sigma-Aldrich) is highly specific for N domain active site, Abz-LFK(Dnp)-OH (Sigma-Aldrich) for C domain active site and Abz-FRK(Dnp)P-OH (Sigma-Aldrich) can be cleaved by both active sites. The reaction mixtures contained 100 mM tris(hydroxymethyl)aminomethane hydrochloride (TRIS HCl, Sigma-Aldrich), 50 mM NaCl, 10 μM ZnCl_2_ and 40 μM Abz-SDK(Dnp)P-OH or 50 μM Abz-LFK(Dnp)-OH or 10 μM Abz-FRK(Dnp)P-OH fluorescent substrate, and desired amount of samples, at pH 7.0. Measurements were performed in black, 96-well plates (Greiner-Bio One) at 37°C, λ_ex_ was 340 nm, λ_em_ was 405 nm. Changes in fluorescence intensities were measured at 4-min intervals in case of domain specific substrates for at least 90 min, and at 1.5-min intervals in case of Abz-FRK(Dnp)P-OH substrate for at least 30 min with a plate reader (NovoStar plate reader; BMG Labtech). Fluorescence intensity values were plotted as a function of reaction time and fitted by a linear regression (GraphPad Prism 5.0). The fit and the data were accepted when *r* ^2^ was >0.95. ACE activity was calculated via the equation:

activity = (*S*/*k*)*D,

where *S* is the rate of observed increase in fluorescent intensity (1/min), *k* is the change in fluorescence intensity upon the complete cleavage of 1 μmol of fluorescent substrate, and *D* is the dilution of the sample. ACE activity is given in units where 1 U is equivalent to the cleavage of 1 μmol of fluorescent substrate in 1 min.

### Partial purification of human serum ACE

Serum samples from a healthy volunteer were ultrafiltered through ultrafiltration devices with a pore size of 100 kDa (Vivaspin 500, Sartorius Stedim Biotech) at 4°C for 6 min at 15,000 *g*. One volume of initial serum samples were diluted to 250-fold in 25 mM HEPES, pH 8.2 (yielding 250 volume of diluted serum samples). Then these diluted serum samples were ultrafiltered until the retained volumes reached the initial volumes of the serum samples (1 volume).

### Expression and purification of recombinant ACE

**Figure 2:**
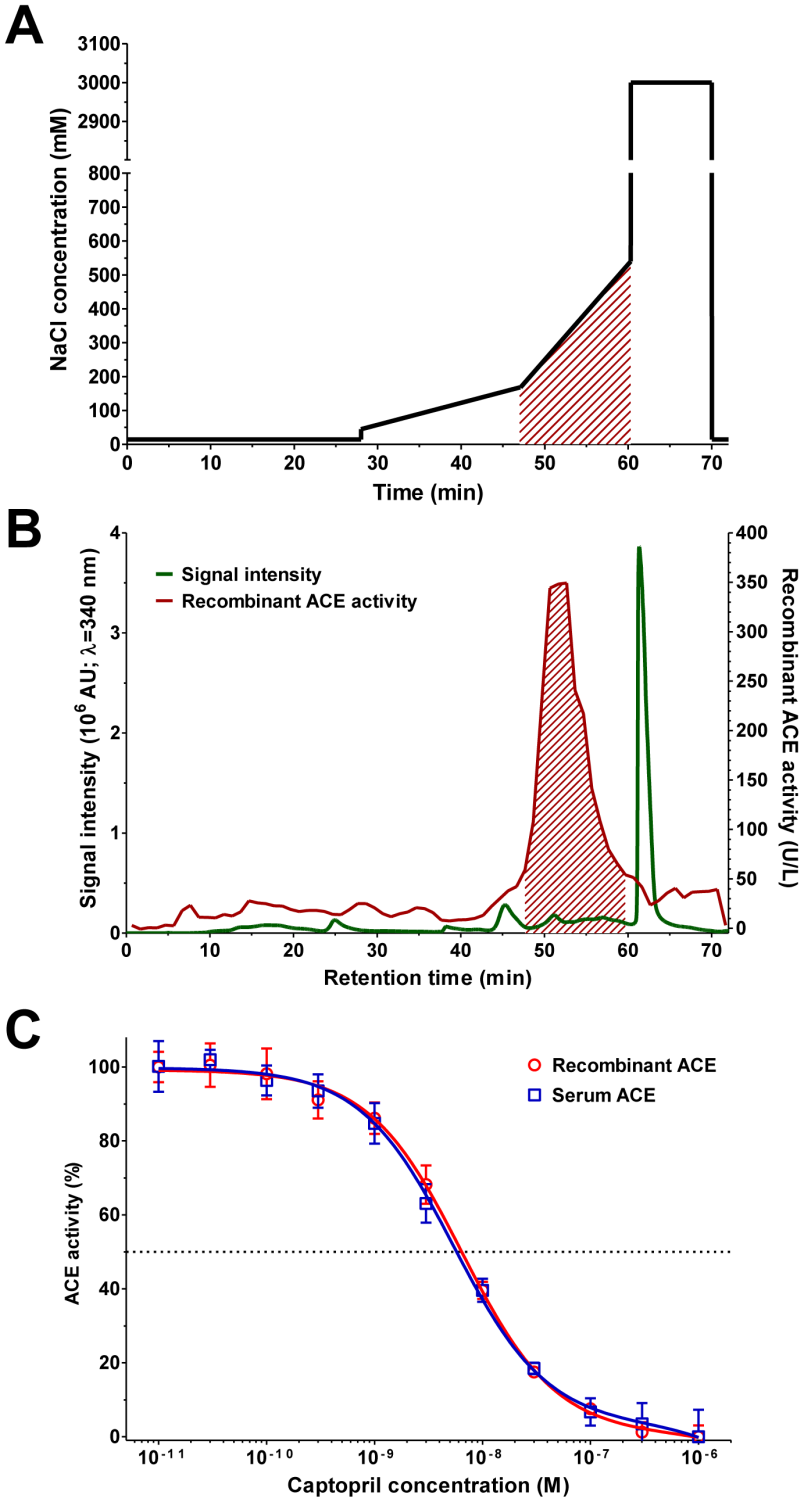
Characteristics of recombinant ACE. Recombinant ACE was produced with a Bac-to-Bac TOPO expression system (Invitrogen) according to the manufacturer's instructions. The pellet of baculovirus infected SF9 insect cells was homogenized in radioimmunoprecipitation assay buffer (RIPA) by sonication. The supernatant was injected onto an anion-exchange column. Column was then washed with a running buffer with different NaCl contents (shown on **A**). The ACE was eluted with a gradually increasing concentration of NaCl from 168 mM to 540 mM (hatched, **A**). Protein concentration was continuously measured (absorbance at 230 nm, green line, **B**) while ACE activity was measured in the collected fractions (300 μL each, red line on Panel **B**). Fractions with at least 50 U/L activity were combined (determined by FAPGG hydrolysis, hatched, **B**). Inhibitory effect of captopril was tested on the purified recombinant and on the serum ACE. Symbols represent the mean, bars are SEM of the three independent determinations.

Recombinant ACE was produced with a Bac-to-Bac TOPO expression system (Invitrogen) according to the manufacturer's instructions. Briefly, a chemically competent *E. coli* strain (Invitrogen) was transformed with an ACE gene containing cDNA plasmid (GeneCopoeia). After antibiotic selection and plasmid isolation, the pFastBac construct containing the ACE coding sequence was transformed into DH10Bac competent *E. coli* (Invitrogen) to generate recombinant bacmid. The bacmid DNA was transfected into the SF9 insect cell line (Invitrogen), in which baculovirus was generated. Further SF9 insect cells were infected with these bacoluviruses. On day 4, the insect cells were centrifuged (1,000 *g*, 10 min, 4°C) and the pellets were washed in PBS to remove the cell culture medium. The pellet was then homogenized in radioimmunoprecipitation assay buffer (50 mM Tris, 150 mM NaCl, 1% Triton X 100, 0.1% SDS, 1% deoxycholate; RIPA buffer) by sonication (Bandelin Electronic). The supernatant was collected by centrifugation (15,000 *g*, 10 min, 4°C), and injected onto an anion-exchange column (Knauer, Biofox Q) in 25 mM HEPES, 15 mM NaCl, pH 8.2. The ACE was eluted with a gradually increasing concentration of NaCl (from 168 mM to 540 mM, hatched, Fig. 2A). ACE activity was measured in each collected fractions (300 μL each), and fractions with at least 50 U/L activity (determined by FAPGG hydrolysis, hatched, Fig. 2B) were combined. Pharmacological properties of the recombinant ACE were compared to human serum ACE. No differences were noted in ACE inhibition by captopril (activity was determined by FAPGG hydrolysis) when endogenous and recombinant ACE were used (Fig. 2C).

### Detection of the molecular interactions of human ACE

#### Crosslinking ACE in the serum

**Figure 3:**
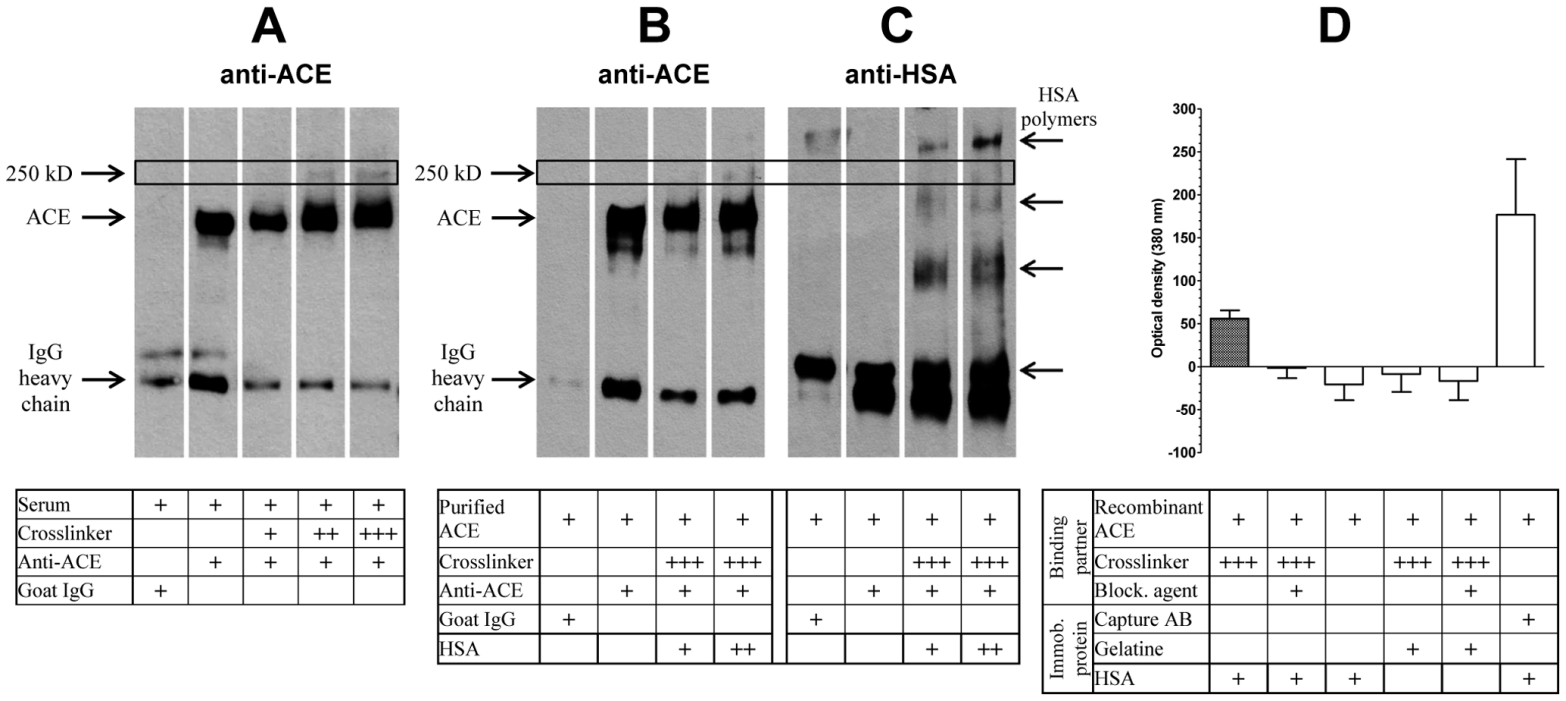
Human serum albumin interacts with the ACE. Human serum samples were incubated with amino (-NH_2_) and carboxyl (-COOH) group-reactive heterobifunctional crosslinkers (succinimidyl[(*n*-maleimidopropionamido)-dodecaethyleneglycol] ester, SM(PEG)_12_, and succinimidyl[(*n*-maleimidopropionamido)-hexaethyleneglycol] ester, SM(PEG)_6_), at concentrations of 1.25, 2.5 and 5 mM (labeled +, ++ and +++, respectively) for 60 min at room temperature. Free amino groups were then blocked by TRIS (50 mM) and the adducts were immunoprecipitated and detected by an anti-ACE antibody (**A**). In some cases the anti-ACE antibody was omitted (first lane, **A**). The apparent molecular masses of ACE, the IgG heavy chain and the crosslinked product are shown on the left (**A**). Similar experiments were performed with purified ACE (100 kDa ultrafiltered serum) and purified HSA (24 and 48 mg/mL, labeled + and ++, respectively). Crosslinked proteins were precipitated by anti-ACE antibody (except for the first lanes, where control goat IgG was added) and detected by anti-ACE (**B**) and anti-HSA (**C**) antibodies. The positions of the IgG heavy chain, ACE and the crosslinked product are shown on the left (**B**), while the positions of the monomeric and multimeric HSA are shown on the right (**C**). Finally, the ACE-HSA interaction was also characterized by ELISA assay (**D**). 96-well plates were coated with HSA (1% in PBS) or with gelatine (1% in PBS) overnight. Bound proteins were incubated with SM(PEG)_12_ and SM(PEG)_6_ (each 625 μM) and recombinant ACE was then added to the wells (26 ng/well). Bars denote means ± SEM of the results of 4 independent experiments performed in triplicate.

Molecular interactions were stabilized by heterobifunctional crosslinkers. First, the interactions of ACE in human serum were tested. Sera were diluted in 6.25 mM HEPES buffer (pH 7.2) and succinimidyl-[(*N*-maleimidopropionamido)-dodecaethyleneglycol] ester (SM(PEG)_12_), and succinimidyl-[(*N*-maleimidopropionamido)-hexaethyleneglycol] ester (SM(PEG)_6_), (both from Thermo Scientific) were then added at concentrations of 1.25 mM, 2.5 mM and 5 mM (labeled +, ++ and +++, respectively in Fig. 3A). The mixture was incubated for 60 min at room temperature in order to stabilize intermolecular interactions, and the functional succinimidyl groups of the crosslinker molecules were then blocked by 50 mM Tris (tris(hydroxymethyl)aminomethane; Sigma-Aldrich) for 30 min at room temperature. The ACE and the crosslinked proteins were then immunoprecipitated. Biotinylated goat anti-human ACE antibody (R&D Systems; 22.6 ng/mL) or goat IgG (22.6 ng/mL; control) and immobilized streptavidin resin (Pierce) were added to the mixture to anchor the crosslinked products to the surface of the resin. The mixture was incubated overnight at room temperature with continuous agitation, after which the resin-bound complexes were washed 5 times with 25 mM HEPES pH 7.2. The immunoprecipitated complexes were then prepared for SDS-PAGE (the resin was boiled for 10 min in 2× concentrated SDS sample buffer (Sigma-Aldrich)). The samples were loaded on a 5–15% gradient gel (Mini-Protean TGX Precast Gel, Bio-Rad Laboratories) and ACE was detected with a goat anti-human ACE antibody at a dilution of 1∶1,000 (R&D Systems), while the secondary antibody was a peroxidase-linked anti-goat antibody at a dilution of 1∶40,000. The blot was developed on green sensitive medical X-ray film (Primax Berlin), using the Western Lightning Plus-ECL reagent (Perkin Elmer Life Sciences).

#### Testing the interaction of serum ACE with purified HAS

The molecular interaction between human serum ACE and HSA was also tested. First, human serum was filtered through a membrane with a cutoff size of 100 kDa (Vivaspin 500; Sartorius Stedim Biotech) to remove endogenous HSA and other low molecular mass proteins but to retain ACE (molecular mass 180 kDa). HSA was obtained from Human BioPlazma, Gödöllő, Hungary. The interaction between ACE and HSA was then tested by crosslinking (5 mM SM(PEG)_6_) in the filtered serum. Reaction mixtures were prepared in the absence or in the presence of 24 and 48 mg/mL HSA (labeled + and ++ in Fig. 3B and 3C, respectively) under the conditions mentioned above. The membranes were probed with the anti-ACE antibody as mentioned above, then stripped (incubation in 1% sodiumdodecylsulfate (SDS, Serva), 20 mM DL-dithiotreitol (DTT, Sigma-Aldrich) and 62.5 mM Tris, pH 7.5 for 20 minutes at 60°C) and re-probed with a biotinylated anti-HSA antibody (Exbio, Praha) as primary antibody in a dilution of 1∶5,000 (60-min incubation) and peroxidase-streptavidin in a dilution of 1∶100,000 (Jackson; 60-min incubation) to visualize the position of HSA.

#### Crosslinking of recombinant ACE and purified HSA

The interaction between ACE and HSA was tested on ELISA plates (Greiner Bio-One). First, HSA (Human BioPlazma, Gödöllő, Hungary, 1% in PBS, 300 μL/well) was added to the wells and incubated overnight at room temperature. The next day, wells were washed 5 times with PBS, SM(PEG)_6_ and SM(PEG)_12_ were added (each 625 μM in PBS, 100 μL/well) and the mixtures were incubated with the surface-bound HSA for 30 min. After thorough washing (at least 5 times with PBS), 100 μL of 260 ng/mL recombinant ACE (R&D Systems) was added to the wells for 1 h, the wells were washed at least 5 times with PBS and the immobilized HSA-linked ACE was detected (Fig. 3D).

### Direct measurement of ACE-catalyzed bradykinin cleavage

Recombinant ACE was incubated with 1 μM bradykinin (Sigma-Aldrich), 10 μM Amastatin (Sigma-Aldrich), 1 μM Z-prolyl-prolinal (Enzo Life Sciences) and 300 mM NaCl in 25 mM HEPES buffer, pH 7.40 in the absence (vehicle) or presence of 40 mg/mL HSA (Human BioPlazma, Gödöllő, Hungary) at 37°C. 5 mM EDTA was added to stop the reaction. Bradykinin peptides were measured after filtering through a filter with a 10 kDa pore size (Vivaspin, Sartorius Stedim Biotech). Analysis was performed with a HPLC technique on a reverse-phase C18 column (Hypersil Gold, Thermo Scientific). Eluent A was 0.01% aqueous trifluoroacetic acid (TFA, Sigma-Aldrich), while eluent B was 0.01% TFA in acetonitrile (Sigma-Aldrich). Bradykinin peptides were separated by using an elution profile with a gradient from 18% acetonitrile to 44.2% acetonitrile. They were detected by a diode array detector at 230 nm and the area under the curve of each bradykinin peptide peek was compared with calibration curves recorded when the purified peptide was tested. The amounts of bradykinin peptides were plotted against the reaction time and fitted by linear regression. The kinetics of bradykinin cleavage was normalized to the background (recombinant ACE plus 1 μM captopril (Sigma-Aldrich)), and compared to vehicle (recombinant ACE without HSA).

### Isometric contractile force measurement on human saphenous vein segments

Saphenous veins (remained from coronary artery bypass graft surgery) were cut into circular segments. The veins were placed in ice-cold physiological buffer solution (containing 110 mM NaCl, 5 mM KCl, 1 mM MgSO_4_, 1 mM KH_2_PO_4_, 5 mM D-glucose, 24 mM NaHCO_3_, pH = 7.4). Rings were then mounted on a dual wire myograph system (DMT 510A; Danish Myotechnology). The organ chamber was filled with oxygenated (10% O_2_, 5% CO_2_, 85% N_2_) physiological buffer solution containing 2.5 mM CaCl_2_, and vein segments were stretched at 15 mN and incubated under isometric conditions for 60 min at 37°C. The viability of the mounted vascular rings was tested with 56 mM KCl and 10 μM norepinephrine. The mounted veins were then washed. The vascular contractile function was tested with 1 μM angiotensin I and II in the presence or absence of 20 mg/mL HSA. At the end of the measurement, the norepinephrine and KCl treatments were repeated in the presence of the angiotensin peptides to confirm the viability of the vascular rings. HSA was also applied together with captopril in some cases.

### Statistical analysis

Statistical analysis was performed with Graphpad Prism 5.0 (GraphPad Software) by paired and unpaired t-tests. Differences were considered to be significant when *p*<0.05.

## Results

The interaction of ACE with its suspected endogenous inhibitor was stabilized by crosslinking amino and carboxyl groups within the interacting proteins (crosslinker spacer arm: 5.3 nm). ACE-containing complexes were then identified by immunoprecipitation and then by using an ACE-specific antibody in Western blotting (Fig. 3A). Besides the 180 kDa band indicative of free ACE, an extra band appeared in the crosslinked samples, with an apparent molecular mass of about 250 kDa (Fig. 3A). The size of the crosslinked product (about 250 kDa) suggested that the interaction partner is about 70 kDa. The most abundant plausible protein with that molecular mass is the human serum albumin (HSA, 66 kDa). This hypothesized interaction (between ACE and HSA) was directly tested with purified ACE and HSA by the same crosslinking technique (Fig. 3B and 3C). The 250 kDa adduct was again observed and positively stained by both anti-ACE (Fig. 3B) and anti-HSA antibodies (Fig. 3C) in Western blot. The interaction between HSA and ACE was further confirmed by ELISA. HSA, gelatin or ACE specific antibody was immobilized on the surface of ELISA plates and the binding of ACE to these surfaces was tested after crosslinking or alternatively when the crosslinking was blocked or the crosslinkers were omitted (controls of specificity). Immobilized ACE (crosslinked or antibody bound) was detected. A high level of crosslinked ACE was detected after successful crosslinking reactions (first bar, Fig. 3D), but not when the crosslinking was blocked (second bar, Fig. 3D) or the crosslinkers were omitted (third bar, Fig. 3D). In contrast with HSA, ACE crosslinking was not observed in gelatin coated plates (fourth and fifth bars, Fig. 3D). Finally, the maximum ACE binding capacity of the surface was estimated by the immobilization of an anti-ACE antibody (last bar, Fig. 3D).

**Figure 4:**
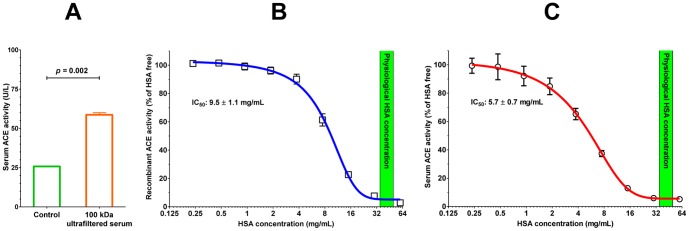
HSA inhibits serum and recombinant ACE. Purification of ACE by filtration through 100±0.3 to 58.6±1.3 U/L (**A**). Effects of HSA were tested on recombinant (**B**) and partially purified ACE (100 kDa ultrafiltered serum; **C**). The ACE activity was measured by FAPGG hydrolysis. Symbols denote means ± SEM of the 3 independent experiments. Data were fitted by nonlinear regression and the calculated IC_50_ values are shown. The physiological HSA concentration range (35–52 mg/mL) in human serum is also indicated by green.

Potential inhibitory effect of HSA was also tested on partially purified ACE from the human sera. Separation of the ACE from the HSA in human sera (by filtration through a filter device with a 100 kDa pore size) resulted in an increase in ACE activity (activities at the lowest dilution in Fig. 4A). HSA also inhibited recombinant human ACE (Fig. 4B) and partially purified serum ACE (Fig. 4C) activities directly, with half maximal inhibitory concentrations (IC_50_) of 9.5±1.1 and 5.7±0.7 mg/mL, respectively.

A common clinical side effect of ACE inhibitory therapy is coughing as a result of elevated bradykinin levels. Effects of 40 mg/mL HSA (saturating concentration in FAPGG hydrolysis, Fig. 4B) were tested on bradykinin hydrolysis by recombinant ACE. HSA only partially inhibited bradykinin breakdown (inhibition by 70.4±11.4%, n = 3, *p*<0.01 compared to vehicle).

**Figure 5:**
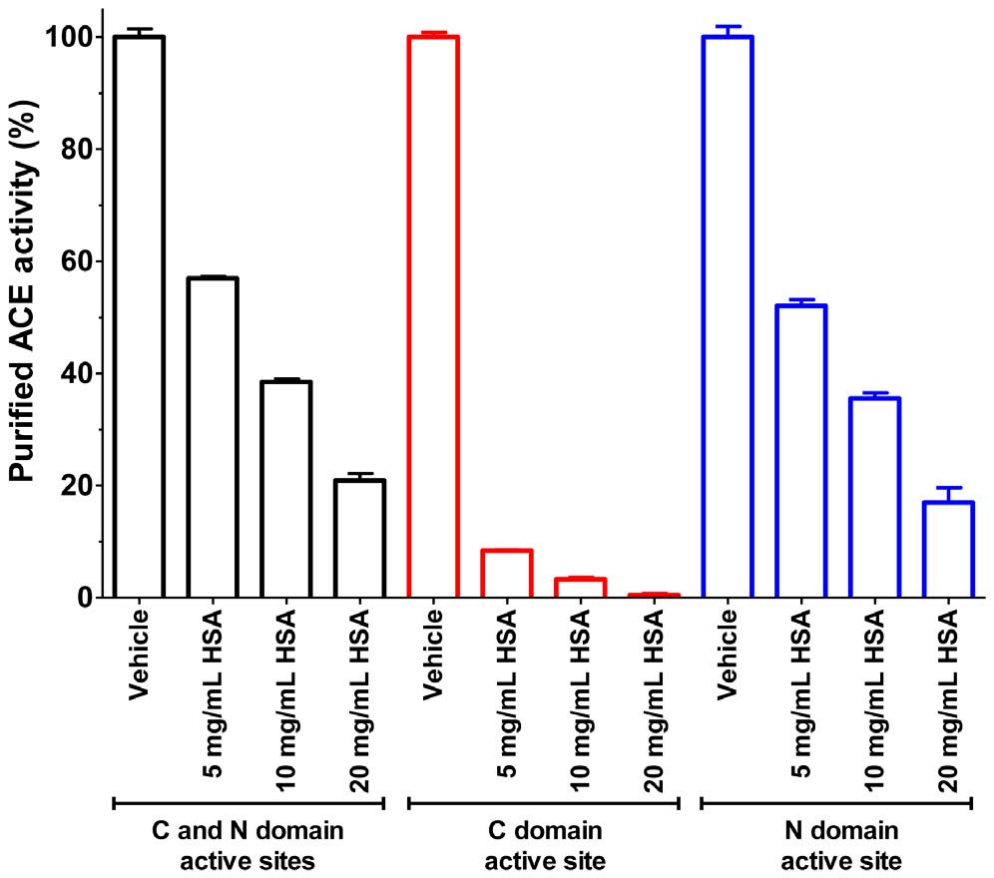
HSA has higher affinity for the C-terminal active site of purified ACE. Inhibition of serum ACE was tested by active site specific fluorescent substrates: Abz-SDK(Dnp)P-OH (blue bars) for the N-terminal active site, Abz-LFK(Dnp)-OH (red bars) for the C-terminal active site. Abz-FRK(Dnp)P-OH (black bars) was used as non-site specific substrate. Bars represent means ± SEM of 3 independent determinations, values are given in the percentage of control (vehicle, without ACE inhibitor).

ACE has two catalytically active sites, which have slightly different substrate specificities. Effects of HSA were tested on these sites by specific fluorescent substrates. It was found that HSA has a higher affinity for the C-terminal active site (Figure 5).

**Figure 6:**
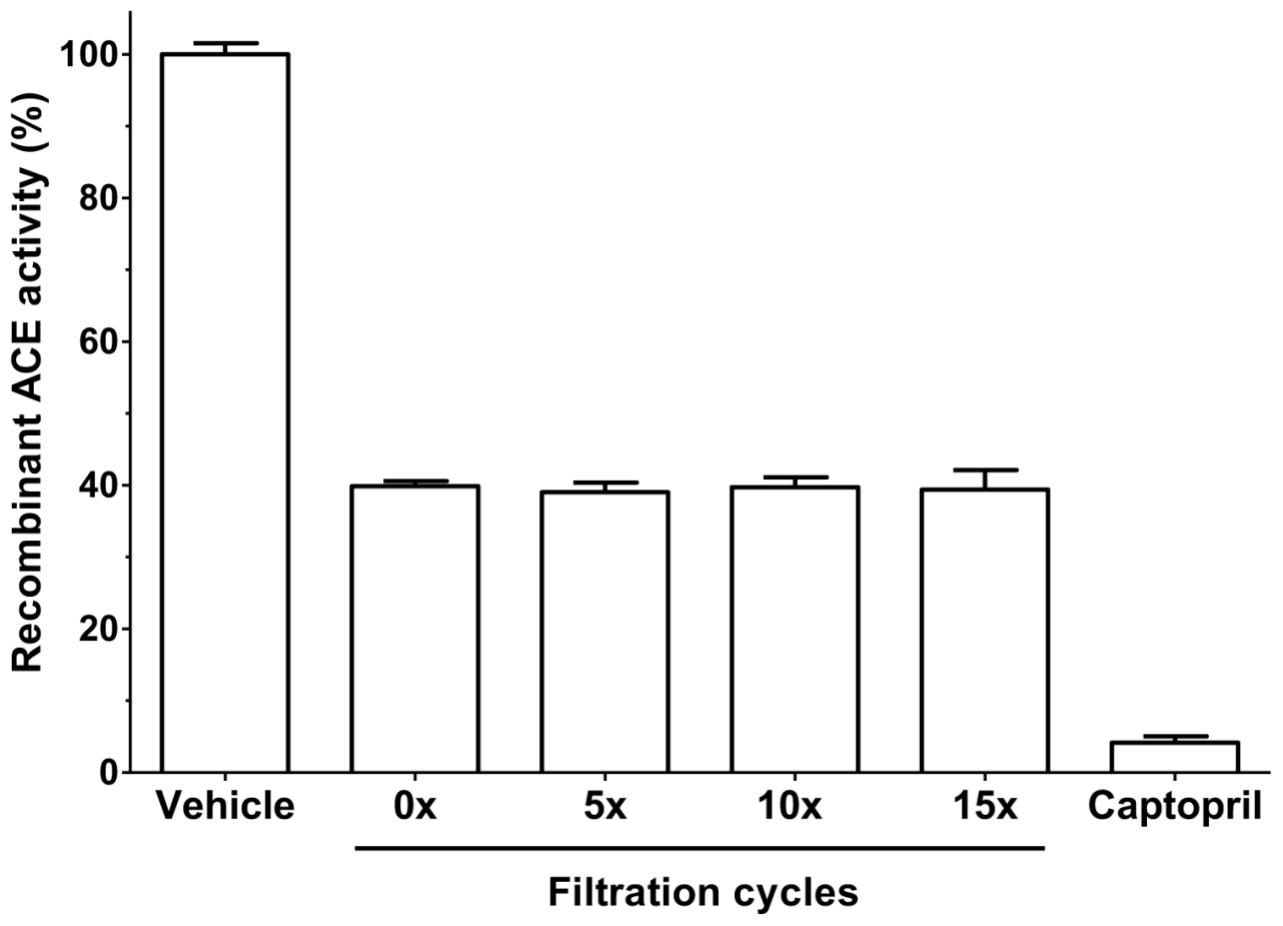
There is no dissociative small molecular weight ACE inhibitor absorbed by the HSA. HSA was diluted to 20/mL in FAPGG reaction buffer and split into 4 fractions with identical volume. The first was used as a control (initial sample, 0 filtration cycles). The HSA was diluted in the other fractions by 10-fold and filtered through 5 kDa pore size membranes until the HSA concentration (and volume) of the retained fractions reached the original 20 mg/mL. This filtration step was repeated by 5, 10 or 15 times as indicated on the figure. The effects of these HSA fractions were tested on recombinant ACE using FAPGG substrate at a final concentration of 10 mg/mL. Maximal ACE activity was determined in the absence of HSA (vehicle). Captopril (1 μM) was also used to estimate the effect of complete ACE inhibition on FAPGG hydrolysis.

An effort was made to test whether HSA or some HSA absorbed dissociative molecules are responsible for ACE inhibition. Inhibitory effect of a half-maximal concentration of HSA (10 mg/mL) was tested before (initial sample) and after filtering via filters with a pore size of 5 kDa (5, 10, 15 filtration cycles) on recombinant ACE activity. No effect of serial filtration was seen, while captopril successfully inhibited the ACE activity (Fig. 6).

**Figure 7:**
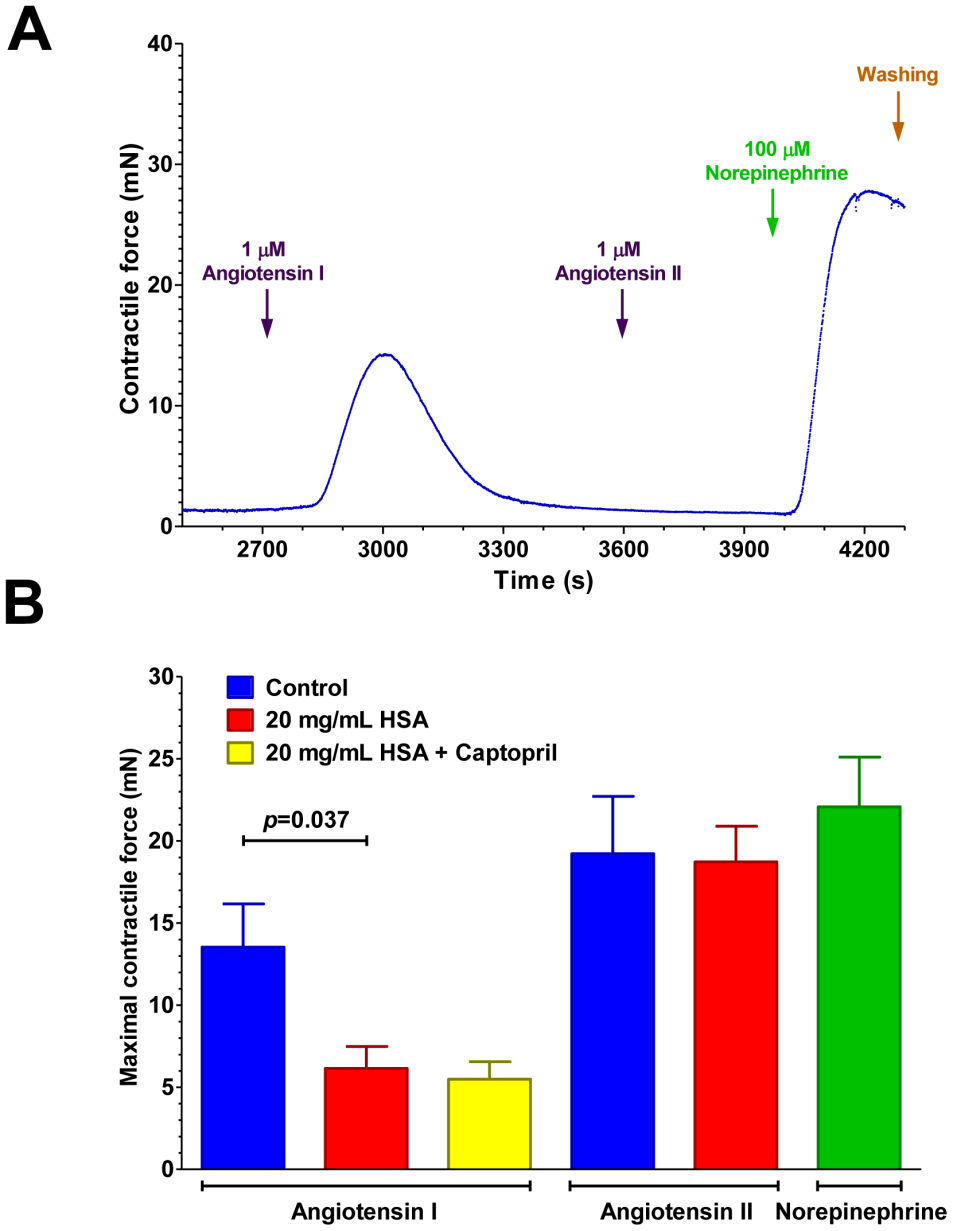
HSA inhibits tissue ACE in human vascular bed. Human saphenous vein rings were mounted on an isometric contractile force measurement setup. Vessels were treated with angiotensin peptides (angiotensin I in **A**) at 1 μM for an extended period of time (without washing). The vascular segments contracted, as indicated by the increase in contractile force, and then relaxed in the continuous presence of angiotensin I. At the end of the experiment, 1 μM angiotensin II was added to confirm desensitization, and the viability of the vessel was finally tested with norepinephrine (100 μM). An individual experiment is illustrated in **A**, force was recorded in every 0.5 s. The effects of HSA were tested on separate vascular segments. Maximal contractile responses are shown on the bar graph (**B**) to angiotensin peptides in the absence (Control, blue) or presence of 20 mg/mL HSA (red), in the presence of 20 mg/mL HSA plus 10 μM captopril (yellow) or norepinephrine (green). Bars denote means ± SEM, significant difference is indicated by the *p* value.

ACE inhibition by HSA was tested on tissue-bound endogenous ACE in human vascular preparations (saphenous vein; representative experiment, Fig. 7A and statistics, Fig. 7B). Angiotensin I or angiotensin II was applied in the absence (Control) or in the presence of 20 mg/mL HSA. The combined effects of 20 mg/mL HSA and captopril (10 μM) was also tested on angiotensin I responses. There were no significant differences in constrictions to angiotensin I and II in the absence of HSA (13.5±2.6 mN, *n* = 7, and 19.2±3.5 mN, *n* = 7 respectively, Fig. 7B). Angiotensin I mediated contractions decreased to 6.1±1.3 mN (*n* = 6, *p* = 0.037) in the presence of HSA while angiotensin II evoked contractions were not affected (18.7±2.2 mN, *n* = 7, Fig. 7B). Addition of captopril to 20 mg/mL HSA did not affect vascular constriction to angiotensin I compared to HSA alone (5.5±1.1 mN, *n* = 8, Fig. 7B).

Angiotensin peptide evoked constrictions (unlike norepinephrine evoked ones) were transient. Contractile responses diminished within 10–15 min in the continuous presence of angiotensin peptides (Fig. 7A) and application of angiotensin II (1 μM) was without effects in that phase of response (complete desensitization, Fig. 7A), while norepinephrine was able to evoke constrictions (Fig. 7A). The maximal contractile capacity of the blood vessel segments was 22.1±3.0 mN (Fig. 7B) as determined by norepinephrine.

**Figure 8:**
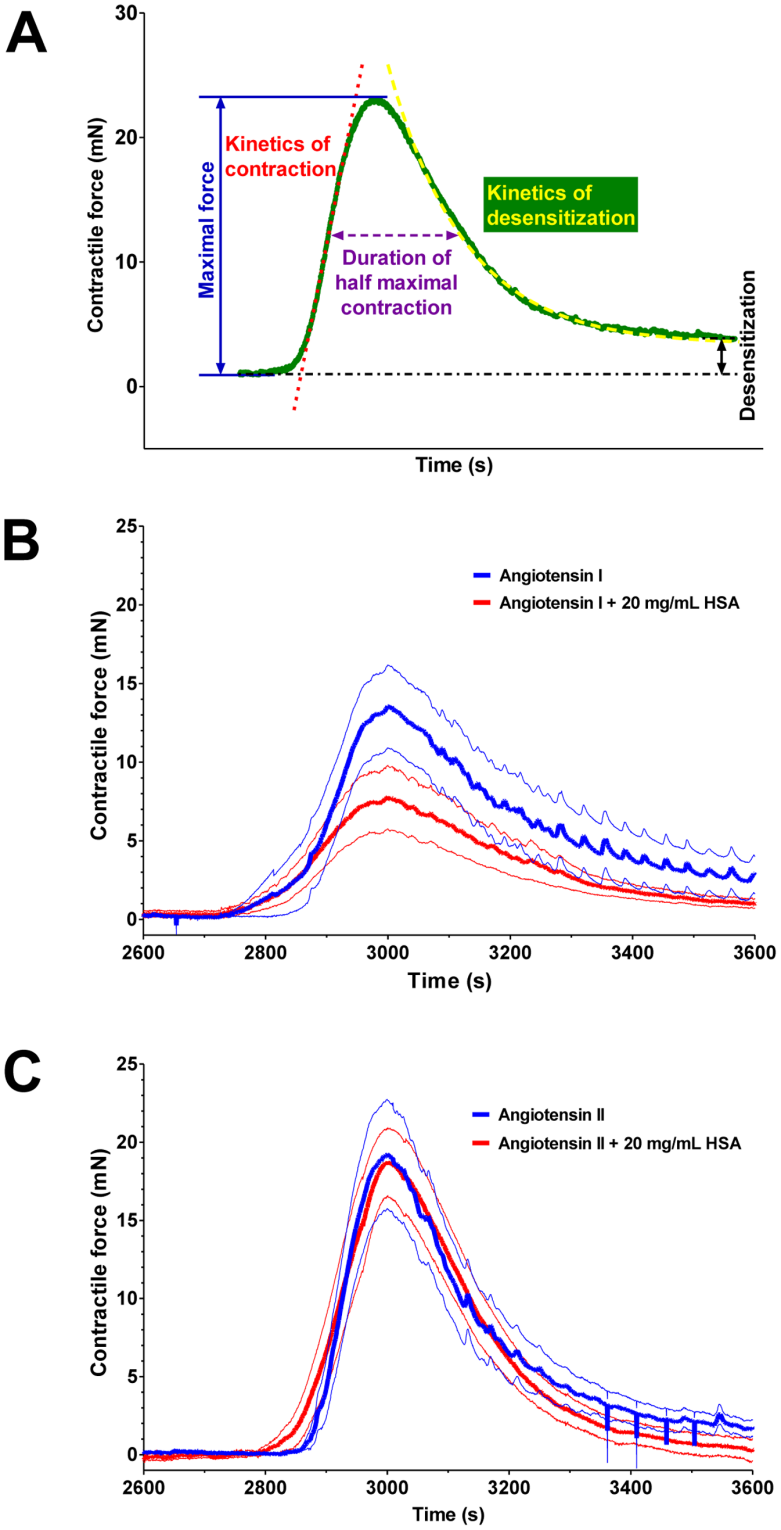
HSA slows down the kinetics and inhibits angiotensin I evoked constrictions in saphenous vein segments. Several parameters of transient contractile response to angiotensin peptides were investigated, including maximal force, kinetics of contraction, duration of half-maximal contraction, kinetics of desensitization and the level of desensitization (**A**). The responses evoked by angiotensin I (**B**) and angiotensin II (**C**) in the absence (blue) and in the presence (red) of 20 mg/mL HSA were recorded on 35 vascular segments from 20 patients. The recorded values were aligned according to the position of the maximal response and the mean ± SEM for each time point was calculated and plotted (mean, thick line, and SEM, thin line; **B** and **C**).

These transient angiotensin responses were investigated in detail (Fig. 8). All of the individual traces of contractile responses were combined to have averaged contractile responses for angiotensin I (Fig. 8B) and angiotensin II (Fig. 8C) in the absence (blue) or presence of 20 mg/mL HSA (red). There were no apparent differences in the angiotensin II responses, while responses to angiotensin I appeared to be affected by the presence of HSA. Motivated by these differences several parameters of the contractile response were determined in each and every individual trace, including the kinetics of the constriction, duration of the half maximal contraction, kinetics of desensitization and the level of desensitization (Fig. 8A), besides to the maximal constriction described before (Fig. 7B).

**Figure 9:**
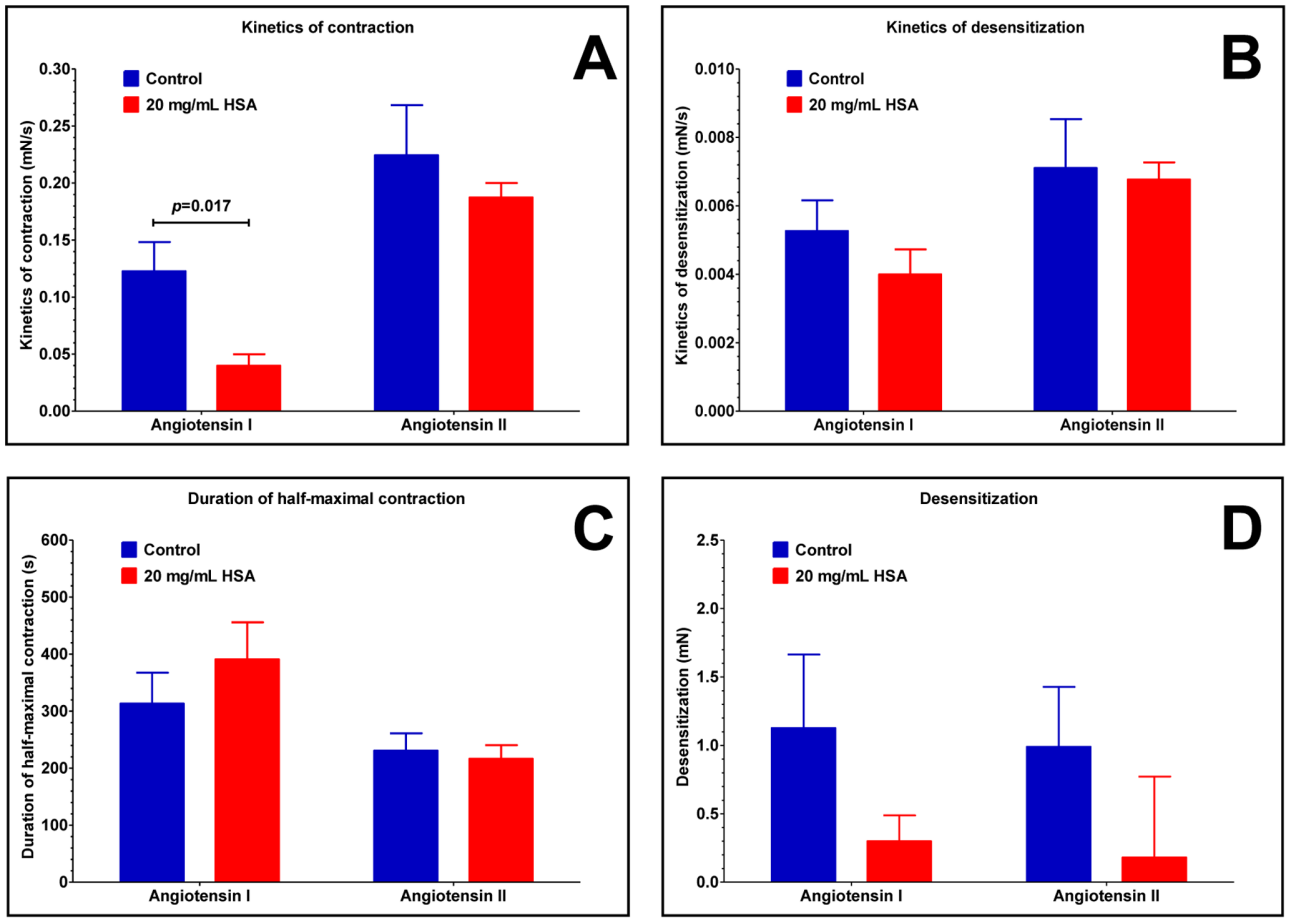
HSA affects the rate of angiotensin evoked force development in human vascular bed. Functional parameters such as kinetics of contraction (**A**), the kinetics of desensitization (**B**), the duration of half-maximal contraction (**C**), and the level of desensitization (**D**) were determined upon angiotensin contractions. Experiments were performed in the absence (blue) or presence (red) of 20 mg/mL HSA. Bars denote means ± SEM. Significant difference is shown by the *p* value.

The kinetics of constriction was about 3-fold slower in the presence of HSA than in its absence (40±10 μN/s, *n* = 6; 123±30 μN/s, *n* = 7; *p* = 0.017; Fig. 9A). In contrast, the presence of HSA had no significant effect on the kinetics of desensitization (Fig. 9B), the duration of the half-maximal contraction (Fig. 9C) or the level of steady-state desensitization (Fig. 9D) in response to angiotensin I treatment. HSA displayed no significant effect on the angiotensin II-evoked responses (Fig. 9A–D).

## Discussion

The existence of endogenous ACE inhibitors were hypothesized before our work and being proven in our accompanying paper [23]. In particular, it was reported earlier that small (<10 kDa) molecular weight molecules can inhibit ACE in 1979 [16]. Several more inhibitors has been suggested afterward [17]–[20]. Some efforts were also made to isolate inhibitory molecules associating with ACE in the rat lung [21] or in human sera [22]. There is also information about an indirect inhibition of ACE by a tissue factor [26].

Interestingly, we did not find evidence of small molecular weight ACE inhibitors in human sera [23], although they have been described previously [16]–[18]. We did not detect any effect of <50 kDa serum filtrates on ACE activity [23]. One of the most probable reasons for interpersonal variances can be the diet of the individuals. It has been reported that dietary factors, such as bovine alphaS2-casein [27] or components of the honey [28] can have ACE inhibitory activities. These data suggests that diet rich in milk products or honey may result in the appearance of small molecular weight inhibitors. This is an important issue (regulation of ACE activity by dietary factors) which needs to be tested in future studies. An alternative explanation is that the observed small molecular weight inhibitors described in previous studies are the products of the degradation of large molecular weight ACE inhibitors, such as HSA [29], [30] and that this degradation did not occur in our samples. Alternatively, the small molecular weight inhibitors were further metabolized and therefore inactivated in our case.

It is an important question whether HSA or some HSA absorbed molecules are responsible for ACE inhibition. The effect of purified HSA on ACE activity suggested that this inhibitory effect is rather linked to the HSA than to any other absorbed molecules. This was further supported by two data of this present manuscript. First, the applied HSA was found to be remarkably pure by mass spectrometry. Second, HSA ability to inhibit recombinant ACE was not affected by up to 15 filtration cycles, suggesting that HSA inhibition is not the result of some dissociative absorbed molecules.

Interestingly, some studies designed to identify dietary components with ACE inhibitory activity lead to the recognition of Acein-1 [29] and albutensin A [30] as tryptic fragments of serum albumin with ACE inhibitory properties. These peptides were synthesized and inhibited ACE, pinpointing HSA regions potentially important in ACE inhibition. Acein-1 was identified as a heptapeptide (Tyr-Leu-Tyr-Glu-Ile-Ala-Arg) spanning the region 138–144 in HSA, while albutensin A is a nonapeptide (Ala-Phe-Lys-Ala-Trp-Ala-Val-Ala-Arg) [30] spanning the region 210–218 in HSA. The existence of these peptides suggest that HSA may have multiple ACE inhibitory sites. Moreover, both peptides have IC_50_ values similar to that determined for HSA here (16 μM for acein-1 [29] and 1.2 μM for albutensin A [30]). Interestingly, the synthetic peptide, which is a single residue longer than acein-1 had a dramatically lower IC_50_ (500 μM versus 16 μM for acein-1) suggesting that position and exposition of these segments of HSA on the surface may have a dramatic effect on their effectiveness.

It become also apparent during the completion of our studies that we are not the first to identify the full length HSA as an endogenous inhibitor of ACE. Klauser et al. has already identified HSA as an endogenous inhibitor of ACE in 1979 [31]. Moreover, they also have identified HSA as a noncompetitive inhibitor, with a Ki value about 3 μM, shown here or in the accompanying paper [23]. The main novelty of the present study is therefore not the identification of HSA as an endogenous inhibitor of ACE, this is only a re-discovery. Nonetheless, we extended our efforts to (1) investigate the effects of HSA on tissue ACE, which is probably has not been done before. In addition (2) we discovered that HSA is probably more sensitive for the C-terminal active site of the serum ACE, and (3) affects angiotensin I and bradykinin hydrolytic activity of ACE differently.

One of the questions regarding our work is the physiological relevance of the findings. Physiological HSA concentrations are several times higher (35–52 mg/mL) than the determined IC_50_ values for HSA (5.7 and 9.75 mg/mL), suggesting complete suppression of ACE activity by HSA, *in vivo*. Moreover, ACE inhibition by HSA were tested on human sera and human blood vessels. Our data suggest that ACE activity is significantly suppressed as long as the HSA concentration is at least ∼30 mg/mL [32]. Nonetheless, the HSA concentration may be lower in conditions associated with protein malnutrition and liver failure, among others. Our findings tend to indicate that under these conditions the infusion of HSA may increase the inhibition of ACE. In accordance with this, it has been found, that the postoperative infusion of HSA frequently evokes hypotension in patients receiving ACE inhibitor therapy [33].

ACE inhibitor drugs are particularly effective in cardiovascular diseases, pinpointing ACE as a major angiontensin I converting enzyme, *in vivo*. HSA was identified here as a major inhibitor of circulating ACE, suggesting that ACE inhibitor drugs are probably not acting on the circulating ACE, since it has already been suppressed by the physiological concentration of the endogenous HSA. ACE inhibitor drugs should therefore target a tissue-bound ACE population. HSA inhibited angiotensin I conversion in the vascular tissue, resulting in a slower and lower maximal angiotensin I mediated constriction in human vascular bed. This effect of HSA was independent of angiotensin II evoked constrictions, suggesting a direct effect on tissue bound ACE, but the efficacy of HSA at 20 mg/mL appeared to be lower in the case of tissue-bound ACE than that is for serum ACE. In addition, captopril (an ACE inhibitory drug) was not more effective than HSA in the same assay (even applied at high concentrations), suggesting that either captopril is not a complete antagonist at vascular ACE or alternatively, angiotensin I responses were not exclusively mediated by ACE in this location.

**Figure 10:**
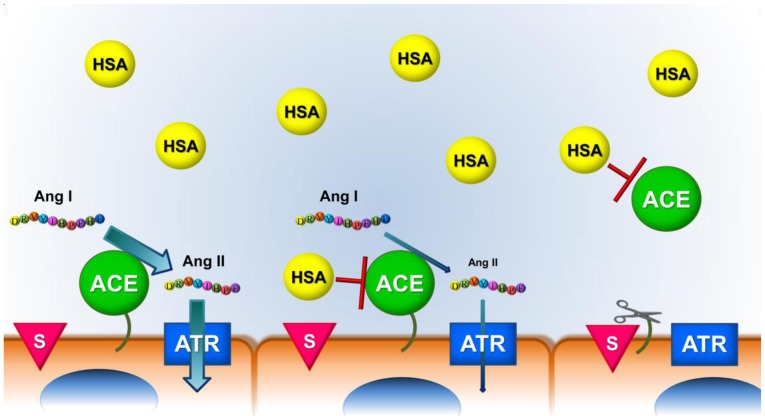
Hypothetical model for the effects of human serum albumin (HSA) on the angiotensin I converting enzyme (ACE) activity. We found a high degree of serum ACE inhibition by physiological concentrations of HSA. This may provide a mechanism for suppressing the circulating ACE, confining angiotensin I conversion to the tissues. Vascular tissue-bound ACE was also found to be inhibited by HSA, *in vitro*. However, ACE inhibitor drugs are markedly effective in hypertension and heart failure. Since serum ACE is suppressed by HSA, ACE inhibitor drugs can probably not inhibit more effectively this ACE population. This can only be explained by the hypothesis that HSA does not uniformly inhibit tissue-bound ACE in the human body. Some of tissue-bound ACE can be inhibited by ACE inhibitor drugs over the inhibition provided by HSA.

Our data provide evidence that endogenous HSA suppresses serum ACE. This can be a mechanism to suppress circulating ACE mediated conversion of angiotensin I, which can lead to systemic (nonspecific) effects (Fig. 10). On the other hand the high level of endogenous ACE inhibition by physiological concentrations of HSA suggests that this circulating ACE probably can not be further inhibited by ACE inhibitory drugs. It appears that an other (tissue-bound) ACE pool is inhibited by these drugs, on which HSA mediated inhibition is not as prominent. One of our novel messages is that ACE activity measurement can mislead in terms of ACE concentration (for example the presence of HSA in the samples result in lower activity values), but it also helps to identify the most likely sources of tissue ACE which is probably not fully inhibited by HSA, *in vivo*. Cushman and Cheung identified the lung as one of the primary source of active ACE [34], pinpointing this location when one is looking for HSA resistant ACE.

We hypothesize that HSA does not inhibit all forms of ACE similarly (Fig. 10). According to this hypothesis ACE in some tissues has lower affinity for HSA or has higher local concentration (expression level) than that is in the serum. HSA mediated inhibition is therefore only limited in these locations, while ACE inhibitory drugs can potently inhibit these enzymes. Under these conditions HSA may inhibit somewhat local ACE activity, but it can be further inhibited by these ACE inhibitor drugs. This is in accordance with a recent consensus report [35] stating that “the plasma ACE represents only a small proportion of the body's total ACE activity, therefore its role is thought to be minimal”. As a matter of fact we have provided a mechanism for this “minimal contribution” by showing HSA mediated suppression of plasma ACE.

An other implication of the high degree of inhibition of serum ACE by HSA is that ACE mediated angiotensin I to angiotensin II conversion can be a rate limiting step in the renin-angiotensin-aldosterone system (RAAS). It is in accordance with the co-existence of both angiotensin I and angiotensin II in the circulation in a comparable level [36], suggesting that the rate of angiotensin I generation (by renin) and conversion (by ACE) are not much different, albeit angiotensin I can not be present at such concentrations without the activation of the renin. Nonetheless, this high degree of ACE inhibition by HSA suggests that the processes responsible for the elimination of the angiotensin II can play a significant role in the determination of local angiotensin II levels. The clinical relevance of this hypothesis was shown in an accompanying paper [37].

There are at least three perspectives which need to be addressed by further studies. First, our results additionally point to new pharmacological strategies for the design of novel ACE inhibitors which could possibly stabilize the interaction between ACE and HSA, thereby resulting in ACE inhibition for a prolonged period. Second, the observed inhibitory effects of HSA on ACE in the sera suggest the existence of a physiological ACE suppressing system. HSA may theoretically stabilize ACE activities at a very low level irrespectively of the actual ACE concentration. In this process, the HSA is present in excess and the ACE associates and being inhibited with the excess HSA even if there are slight differences in ACE concentrations (similarly to hydrogen ions (analogous to ACE here) and buffers (analogous to HSA here)). This has been addressed by an accompanying clinical paper [32]. Third, physiological ACE activities may be very low and angiotensin II availability may be also controlled by its elimination (metabolism), besides to its synthesis. This hypothesis can be tested by studying ACE2 levels in various cardiovascular diseases as was proven in an accompanying paper [37], such as hypertension and heart failure, where ACE inhibitors are particularly effective.
